# Cyclization strategies of meditopes: affinity and diffraction studies of meditope–Fab complexes

**DOI:** 10.1107/S2053230X16007202

**Published:** 2016-05-23

**Authors:** Krzysztof P. Bzymek, Yuelong Ma, Kendra A. Avery, David A. Horne, John C. Williams

**Affiliations:** a Department of Molecular Medicine, Beckman Research Institute of City of Hope, 1710 Flower Street, Duarte, CA 91010, USA

**Keywords:** meditope, monoclonal antibody, X-ray crystallography, surface plasmon resonance

## Abstract

Recently, a unique binding site for a cyclic 12-residue peptide was discovered within a cavity formed by the light and heavy chains of the cetuximab Fab domain. In order to better understand the interactions that drive this unique complex, a number of variants including the residues within the meditope peptide and the antibody, as well as the cyclization region of the meditope peptide, were created. Here, multiple crystal structures of meditope peptides incorporating different cyclization strategies bound to the central cavity of the cetuximab Fab domain are presented. The affinity of each cyclic derivative for the Fab was determined by surface plasmon resonance and correlated to structural differences. Overall, it was observed that the disulfide bond used to cyclize the peptide favorably packs against a hydrophobic ‘pocket’ and that amidation and acetylation of the original disulfide meditope increased the overall affinity ∼2.3-fold. Conversely, replacing the terminal cysteines with serines and thus creating a linear peptide reduced the affinity over 50-fold, with much of this difference being reflected in a decrease in the on-rate. Other cyclization methods, including the formation of a lactam, reduced the affinity but not to the extent of the linear peptide. Collectively, the structural and kinetic data presented here indicate that small perturbations introduced by different cyclization strategies can significantly affect the affinity of the meditope–Fab complex.

## Abstract 2

An overview of cyclization strategies of a Fab-binding peptide to maximize affinity.

## 1. Introduction   

**Figure 1:**
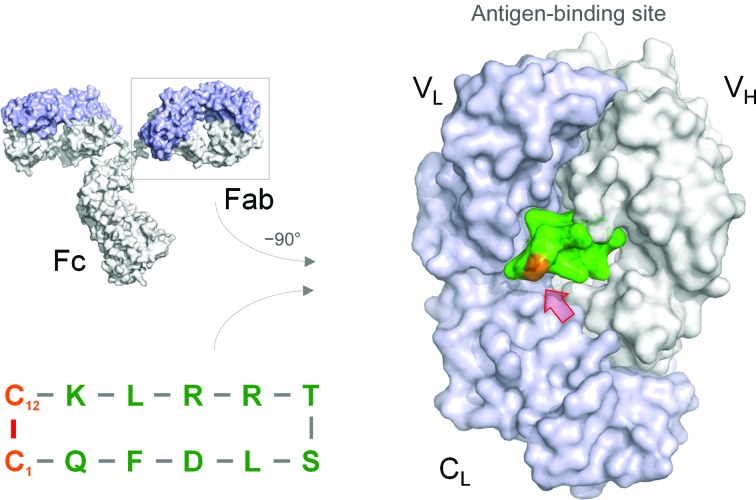
Fab–meditope interface. The light chain is shown in light blue, the heavy chain in dark blue and the meditope in green; the linker residues subjected to mutagenesis are highlighted in orange (highlighted by a red arrow).

Monoclonal antibodies (mAbs) are renowned for their target specificity and favorable therapeutic properties. Owing to these properties, mAbs have become one of the most, if not the most, re-engineered biologics to date. These engineering efforts not only include the humanization and maturation of mAbs to improve their affinity for the antigen, but also the incorporation of unique residues to conjugate cytotoxins, the alteration of protein–protein surfaces to create bispecifics, the fusion of biologics to elicit an immune function, *etc*. In our efforts to add functionality to mAbs, we discovered an un­anticipated peptide-binding site within cetuximab (Fig. 1 ▸; Donaldson *et al.*, 2013 ▸), a chimeric anti-EGFR monoclonal mAb used to treat colorectal and head-and-neck cancers (Huang *et al.*, 1999 ▸; Van Cutsem *et al.*, 2009 ▸; Herbst *et al.*, 2001 ▸). We observed in these studies that the presence of the peptide, which we named the meditope, did not affect the affinity of cetuximab for EGFR. We further demonstrated that we could graft the meditope-binding site onto trastuzumab and demonstrated that neither the grafting nor the presence of the meditope affect antigen affinity. As such, we understood that this binding site represents a unique receptor that could be used to deliver toxins to treat disease or imaging agents to diagnose disease.

In order to modulate the affinity of the interaction and better understand the individual interactions, we have solved multiple X-ray crystal structures of variant meditope–Fab complexes. In this paper, we have focused on different cyclization strategies of the meditope as alternatives to the original disulfide-bonded meditope.

## 2. Materials and methods   

### 2.1. Peptide synthesis   

Meditope peptides were synthesized at the Synthetic and Biopolymer Chemistry Core, City of Hope, Duarte, California, USA or by CS Bio Co., Menlo Park, California, USA. Specifically, standard solid-phase N-αFmoc chemistry was used to synthesize meditope derivatives using a CS136XT peptide synthesizer (CS Bio). Lactam peptides were synthesized starting from Fmoc-Asp(Wang resin LL)-Oall. After cleavage of the peptides from resin using reagent K (TFA:water:phenol:thioanisole:EDT in a 82.5:5:5:5:2.5 ratio), the crude peptides were collected by precipitation from cold ether. For disulfide-linked meditopes, a further oxidation using either 20% DMSO in ammonium acetate buffer pH 6 or iodine was performed. All peptides were purified using reverse-phase HPLC (Agilent 1200 system with Agilent Prep-C18 column; 21.2 × 150 mm, 5 µm) with a water (0.1% TFA)/acetonitrile (0.1% TFA) solvent system. All peptides were characterized by mass spectrometry.

### 2.2. Fab purification   

Cetuximab Fab (Eli Lilly and Company) was prepared by overnight digestion with papain (37°C) followed by reverse purification on a Protein A column (GE Healthcare) (Donaldson *et al.*, 2013 ▸). Unbound protein was further purified using Sephadex 75 26/60 (GE Healthcare) followed by concentration and buffer exchange using an Amicon YM10 membrane (Millipore). Concentrated cetuximab Fab (8–13 mg ml^−1^) was stored at 4°C in 10 m*M* Tris pH 8.0, 10 m*M* NaCl, 1 m*M* EDTA until needed. SDS–PAGE was used to verify the purity of the protein.

### 2.3. Protein crystallization   

Apo cetuximab Fab and complexes with meditope variants were crystallized by the hanging-drop vapor-diffusion method. In a typical experiment, the Fab was mixed with excess meditope (1:10 to 1:20 molar ratio of cetuximab Fab:meditope) and precipitant was added to give a final ratio of 1:1 protein–meditope:precipitant. The precipitant solution consisted of 0.1 *M* Na_2_HPO_4_, 0.1 *M* citric acid, 0.3–0.5 *M* K_2_HPO_4_, 1.5–1.8 *M* NaH_2_PO_4_.

### 2.4. Structure determination   

Crystals of complexes of cetuximab with meditopes were passed through 0.1 *M* Na_2_HPO_4_, 0.1 *M* citric acid, 0.4–0.5 *M* K_2_HPO_4_, 1.6–1.7 *M* NaH_2_PO_4_, 10–15% *meso*-erythritol and 0–5% DMSO and cooled in a cryostream. Diffraction data were collected on a Rigaku MicroMax-007 HF generator with an R-AXIS IV^++^ detector at 100 K and were processed with *XDS* (Kabsch, 2010 ▸).

The structure of each complex was determined by molecular replacement (with *MOLREP* from the *CCP*4 suite; Vagin & Teplyakov, 2010 ▸; Winn *et al.*, 2011 ▸) using a single Fab from the published structure (PDB entry 4gw1; Donaldson *et al.*, 2013 ▸) as the search model. An initial round of rigid-body refinement was followed by restrained refinement using *PHENIX* (Adams *et al.*, 2010 ▸); the model was modified prior to refinement by adding random coordinate shifts (r.m.s of 0.5–1 Å). After an initial round of restrained refinement, the meditope from the structure with PDB code 4gw1 was added to the model. The model of the complex was then iteratively modified using *Coot* (Emsley *et al.*, 2010 ▸), followed by cycles of refinement using *PHENIX*. Default parameters and target weights defined by *PHENIX* were used, with TLS refinement and/or weight optimization to ensure convergence. Restraints for unnatural amino acids were generated using *PRODRG* (Schüttelkopf & van Aalten, 2004 ▸) or *eLBOW* (Adams *et al.*, 2010 ▸) and modified if necessary. Water molecules were added in the later stages of refinement using *PHENIX* with default parameters, followed by inspection of the electron-density maps in *Coot*. Data-collection and model statistics are presented in Table 1 ▸. *F*_o_ − *F*_c_ OMIT maps were calculated using *PHENIX* with default settings (Supplementary Fig. 1 ▸).

All structures of cetuximab Fab–meditope complexes have been deposited in the RCSB PDB (http://www.rcsb.org) with the following codes: (AcN)CQFDLSTRRLRCGGSK (long meditope, disulfide-linked), 5icx; (MPT)QFDLSTRRLKC (mercaptopropionic acid–cysteine linker), 5id1; SQFDLS­TRRKLS (linear peptide), 5icy; (AHA)QFDLSTRRLK (aminoheptanoic acid linker), 5id0; AQFDLSTRRLKA (di-β-alanine linker), 5esq; GQFDLSTRRLKG (diglycine linker), 5icz; (AcN)CQFDLSTRRLKC(Am) (N-terminally acetyl­ated, C-terminally amidated meditope, disulfide-linked), 5hpm; CQFDLSTRRLKC(Am) (C-terminally amidated meditope), 5hyq.

### 2.5. Surface plasmon resonance   

All experiments were performed on a GE Biacore T100 instrument (GE Healthcare). Cetuximab IgG ligand was amine-coupled to CM5 chips using acetate buffer pH 5.5 at densities suitable for kinetics experiments with peptide analytes (5000 RU). Analytes were prepared in GE buffer HBS-EP+ [10 m*M* HEPES pH 7.4, 150 m*M* NaCl, 3 m*M* EDTA, 0.05%(*v*/*v*) surfactant P20]. Kinetics experiments were carried out at a flow rate of 30 µl min^−1^ using HBS-EP+ as both running and regeneration buffer. Experimental data were processed using the *Biacore T100 Evaluation* software v.2.0.1. Purified peptides were dissolved in water and extensively dialyzed to remove any residual TFA. Peptide concentration was assessed by *A*_280_ measurements when possible and was estimated for non-disulfide or Trp/Tyr containing peptides using the formula
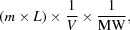
where *m* is the lyophilized peptide mass, *L* is the average percentage loss after dialysis obtained from peptides with measureable *A*_280_, *V* is the final volume of peptide post-dialysis and MW is the molecular weight of the peptide. *L* was calculated from several peptides with measurable absorbance at 280 nm by using the mass before dialysis to calculate the number of moles of peptide present and comparing this number with the calculated number of moles of peptide after dialysis based on *A*_280._ The amount of peptide lost was calculated based on optical absorbance using ten meditope variants (using the calculated tyrosine or disulfide absorbance). The average loss was 53% of the initial mass, with a standard deviation of 38%. This loss is owing to incorrect weight measurements owing to water and TFA and also some loss of peptide during dialysis and tube transfer. Therefore, *L* in the equation above was substituted as 0.5 for the linear, AHA, Gly-Gly and Ala-Ala peptides.

## 3. Results and discussion   

We envision that the meditope interaction can be used to deliver drugs, biologics or imaging agents and/or to cross-link meditope-enabled mAbs bound to cellular receptors to facilitate internalization *in vivo* (Donaldson *et al.*, 2013 ▸). To achieve these goals, we have set out to characterize the interaction to finely tune the affinity for specific applications as well as to address potential issues with serum stability, pharmacokinetics and pharmacodynamics. Here, we focused on the cyclization of the meditope peptide.

The original meditope, selected by phage display, contains two terminal cysteine residues that form a disulfide bond and thus cyclize the peptide (Donaldson *et al.*, 2013 ▸). We observed multiple conformations of the disulfide bond from different crystals using the same peptide, Fab and crystallization conditions. We also observed that the disulfide bond is solvent-exposed in one crystal and buried against Val9 and Ile10 in other crystals. We note, however, that the thermal factors are high compared with the rest of the peptide, consistent with its solvent exposure.

**Figure 2:**
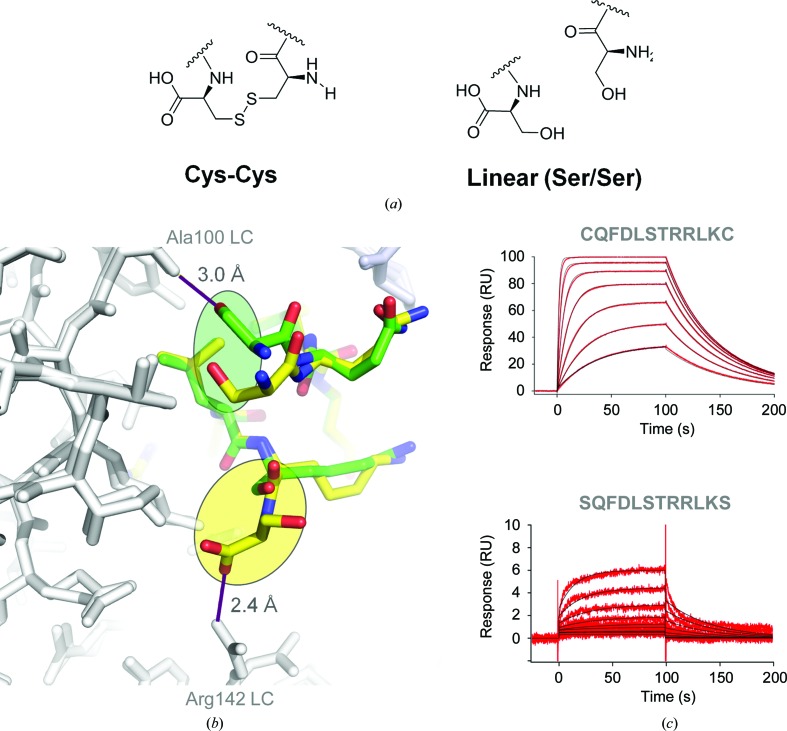
Cyclic *versus* acyclic. (*a*) Replacement of Cys1 and Cys12 with serine as a conservative substitution to create a linear meditope. (*b*) There are two Fab–peptide complexes in the asymmetric unit. Superposition of these complexes indicates flexibility at the N-termini. In one peptide Fab–complex (the peptide with yellow C atoms), the terminal carboxylate makes a favorable salt bridge with the Arg142 guanidinium group from the light chain (LC). In the other peptide–Fab complex (the peptide with green C atoms), the hydroxyl group of Ser1 makes a hydrogen bond to the carbonyl group of Ala100, also of the light chain. However, the electron density is weak and Ser12 could not be built. (*c*) SPR traces of the original meditope and the linear meditope indicate a substantial reduction in binding affinity and an increase in the off-rate.

Based on these observations, we asked whether cyclization is necessary. To address this question, we synthesized a linear variant in which the terminal cysteine residues were replaced by serines (Fig. 2 ▸ *a*). The structure of the complex indicated that that the linear peptide folds in a similar manner when bound to the Fab (Fig. 2 ▸ *b*), but the terminal residues show high thermal factors. In fact, there are two copies of the peptide–Fab complex in the asymmetric unit of this crystal (and all of the structures reported here) and each complex was refined independently of the other (*e.g.* no NCS averaging). As such, we also observe different conformations for the termini of the linear peptide bound to each Fab. In one peptide–Fab complex, the terminal carboxylate of Ser12 makes an electrostatic bond to Arg142 LC (*d*_NH1⋯OXT—C_ = 2.4 Å and *d*_NH2⋯OXT—C_ = 3.5 Å). In the other peptide–Fab complex the electron density is very weak, precluding the building of Ser12 into the model. Moreover, the hydroxyl group of Ser1 in the second complex makes a hydrogen bond to the backbone carbonyl of Ala100 LC (d_OH⋯O=C_ = 3.0 Å). However, as noted, the *B* factors are high, suggesting that the interactions are weak.

The remaining residues of the linear peptide, specifically 2–11, are nearly superimposable in both complexes (r.s.m.d. = 0.14–0.15 Å) and superimpose well on the original meditope peptides (r.s.m.d. = 0.44–0.77 Å). Despite recapitulating many of the original interactions and an extensive hydrogen-bonding network, the overall net affinity of the linear meditope is substantially weaker than that of the original meditope, *K*_d_ = 8.7 µ*M* *versus* *K*_d_ = 170 n*M*, respectively (Table 2 ▸). Of note, the SPR experiments were conducted at a neutral pH, where electrostatic repulsive forces between the two charged, deprotonated carboxylate residues (Ser12⋯Glu105) may be stronger than in the crystal (grown at pH ∼5.5). Weaker binding of the linear peptide is likely to reflect an entropic penalty.

**Figure 3:**
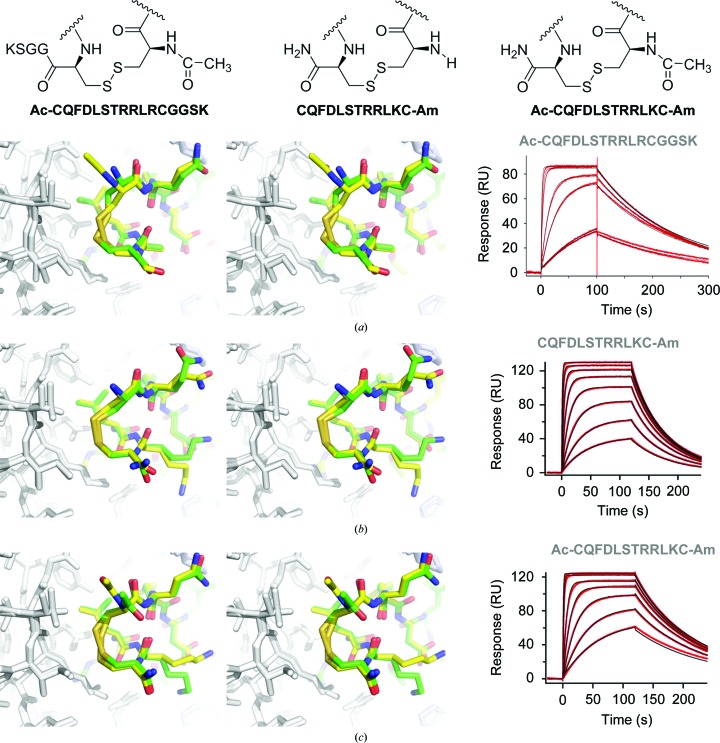
Terminal modifications. Each Fab–peptide complex was superimposed and is shown in stereo with the corresponding SPR trace. (*a*) Long meditope, (*b*) amidated, (*c*) acetylated and amidated complexes. In each case the disulfide packs against Val9 and Ile10 of the light chain. Eliminating charge in each case did not produce substantial structural differences, but did improve the overall affinity.

Given the loss in affinity for the linear peptide, we turned our attention to whether the extension of the cyclic meditope would affect the interaction and whether these alterations would affect the overall affinity. Firstly, we synthesized a modified variant of the meditope termed the ‘long meditope’, with an N-terminal acetylcysteine and an extension at the C-terminus to partially mimic the N-terminally displayed phage peptide [(AcN)CQFDLSTRRLRCGGSK]. N-terminal acetylation also allows future modifications of the peptide (lysine amine). The crystal structure did not reveal significant differences between the long meditope and the original meditope (r.m.s.d. of 0.1 Å for the C^α^ atoms of residues 2–11), with the exception of the cyclization region (cysteines 1 and 12; the r.m.s.d. calculated over 12 C^α^ atoms increased to 0.9 Å), which are in the buried conformation, as discussed above. The remainder of the peptide, GGSK, C-terminal to Cys12 was largely disordered and could not be modeled. However, the C-terminal extension resulted in a modest increase in affinity, *K*_d_ = 110 n*M* for the long meditope compared with *K*_d_ = 170 n*M* for the original meditope (Fig. 3 ▸ *a*).

This modest increase in affinity suggested that the charge on the meditope may affect interaction. Thus, we designed acetylated and amidated variants of the original meditope and measured their binding affinities for cetuximab Fab (Table 2 ▸). The structures are very similar to that of the cetuximab–long meditope crystal structure (Figs. 3 ▸ *b* and 3 ▸ *c*). Both modifications, amidation and acetylation, decreased the dissociation constant to *K*_d_ = 70 n*M*, or a ∼2.3-fold increase in the affinity compared with the original meditope, and showed some improvement over the long meditope. The lack of extension at the C-terminus in addition to reduced charge at the N- and C-termini results in an increased on-rate. The variant which was amidated but not acetylated also produced a slight increase in the affinity, *K*_d_ = 150 n*M*, compared with the initial meditope. We also tested additional N- and C-terminal extensions with the addition of Trp residues (WGGGS-CQFDLSTRRLRC and CQFDLSTRRLRC-GGGSW). We observed that a C-terminal Trp extension produced a binding affinity of 100 n*M*, which is similar to the long meditope C-terminal extension: *K*_d_ = 110 n*M*. The N-terminal Trp extension also improved the binding affinity, *K*_d_ = 63 n*M*, which was similar to the N-terminally acetylated and C-terminally amidated meditope: *K*_d_ = 74 n*M* (Table 2 ▸).

**Figure 4:**
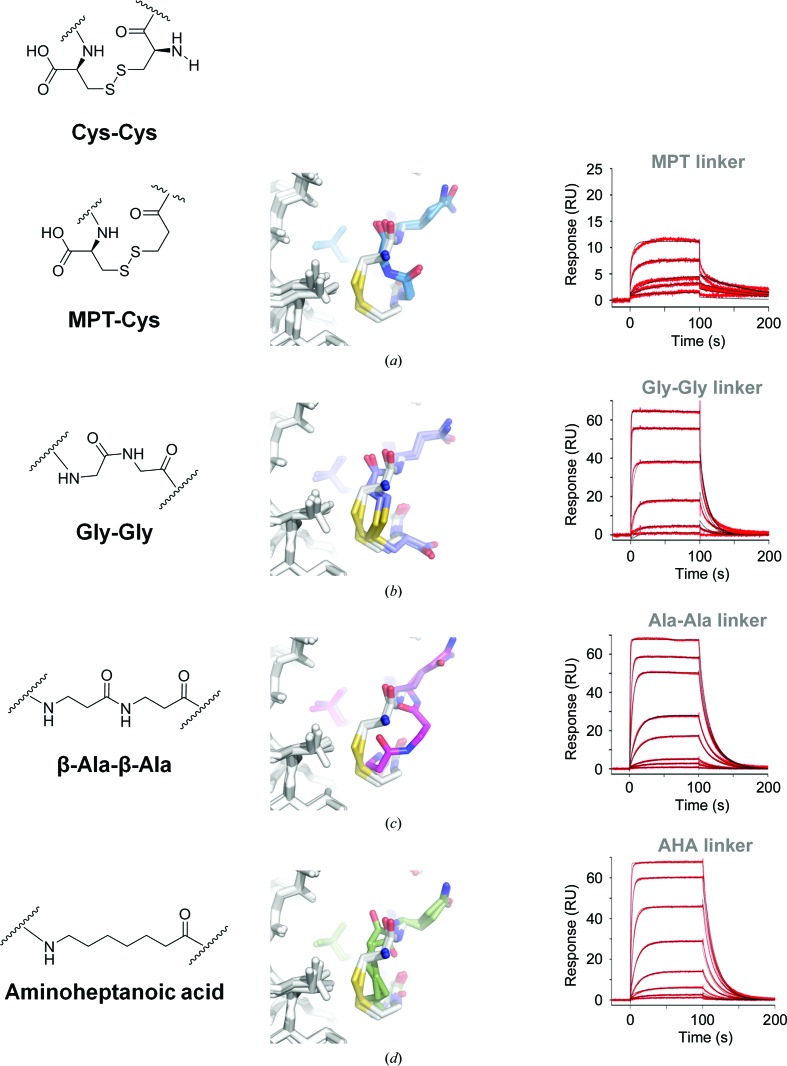
Alternative linking strategies. The left column indicates the modification. The middle column shows the superposition of each Fab–alternative cyclic peptide complex (colored C atoms) on the Fab–acetylated-amidated disulfide peptide (white C atoms). The right column is the corresponding SPR trace. In all cases, the linking atoms are shifted away from the Val9/Ile10 residues and thus do not pack as well. The affinity of the aminoheptanoic acid linker (AHA) was the closest to the original disulfide meditope.

Next, we sought to directly probe the importance of van der Waals interactions. Firstly, we replaced Cys1 with mercaptopropionic acid. This conservative substitution does not change the geometry or the hydrophobic nature of the linker; however, the lack of an amine at the C^α^ position leads to a slight increase in the distance between C^β^ and SG of the mercaptoic acid and CG of Val9 HC of the Fab (by 0.1–0.2 Å compared with the long meditope; Figs. 4 ▸ *a* and 4 ▸ *b*). The off-rate of the MPT-linked peptide is similar to that of the long meditope (0.012 and 0.010 s^−1^, respectively), although there is a ∼4.4-fold decrease in the on-rate for the MPT derivative (Table 2 ▸).

Adding more flexibility to the linker region and substituting all the S atoms with C atoms (diglycine, β-Ala-β-Ala and AHA linkers; Figs. 4 ▸ *b*, 4 ▸ *c* and 4 ▸ *d*, respectively) results in faster off-rates and weaker overall affinity (Fig. 4 ▸, right; Table 2 ▸). Of note, although there is a significant error in the estimation of peptide concentration for AHA-, β-Ala-β-Ala- and Gly-Gly-linked meditopes, the off-rate is independent of the peptide concentration. As the distance between the cyclization region of the meditope and Val9 and Ile10 of the Fab increases, so does the dissociation constant. Flexibility and geometry dictate the special organization of the β-alanine linker, which has the same number of bridging atoms as the disulfide-linked meditope (eight, including the amide N atom of β-Ala1 and the carbonyl C atom of β-Ala12), while the aminoheptanoic acid (AHA) linker is one atom longer and the diglycine linker is two atoms shorter. As expected, the shortest linker (Gly-Gly) makes the least favorable hydrophobic interactions with Val9 and Ile10, with the shortest distance being ∼4.7 Å for C^α^ of Gly12 and CD of Ile10. In comparison, the respective distances for the long meditope are much shorter (3.2–3.6 Å for C^α^ and SG of the cysteines, CD and CG of Ile10 and CG of Val9).

**Figure 5:**
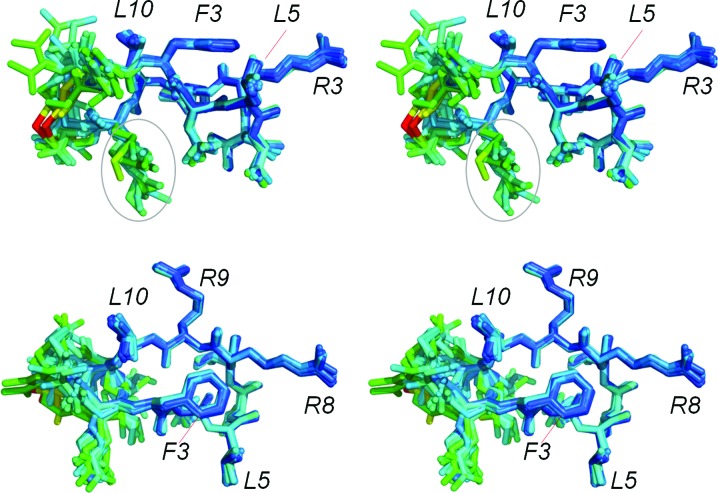
Superposition of all meditope peptide variants. Superposition of each peptide variant (colored by thermal factor) from each complex indicates that the ‘core’ residues, amino acids 3–10, are effectively not perturbed by the cyclization strategies.

Collectively, the binding and structural studies highlight the importance of the cyclization strategy for optimal meditope binding. The acetylated/amidated meditope and the N-terminal Trp extension with disulfide linkers exhibited the lowest *K*_d_, with slower off-rates and higher affinities. We speculate that the increased atomic radius of the S atoms is responsible for the favorable hydrophobic interaction with Val9 and Ile10 of cetuximab Fab (Nagano *et al.*, 1999 ▸). As expected, the affinity decreased with increasing distance between the linker and the Fab (Cys-Cys > MPT-Cys > AHA ≃ β-Ala-β-Ala > Gly-Gly > linear peptide), underscoring the importance of van der Waals interactions in this region (Fig. 5 ▸). Further modification of the linker region by varying the number of methylene groups and/or adding rigid elements (double bonds) or incorporating a thiol ether may result in further improvement of the binding affinity.

## Supplementary Material

PDB reference: cetuximab Fab with AQFDLSTRRLKA meditope, 5esq

PDB reference: with (AcN)-CQFDLSTRRLKC-(Am) meditope, 5hpm

PDB reference: with CQFDLSTRRLKC-(Am) meditope, 5hyq

PDB reference: with (AcN)-CQFDLSTRRLRCGGSK meditope, 5icx

PDB reference: with SQFDLSTRRLKS meditope, 5icy

PDB reference: with GQFDLSTRRLKG meditope, 5icz

PDB reference: with (AHA)QFDLSTRRLK meditope, 5id0

PDB reference: with (MPT)QFDLSTRRLKC meditope, 5id1

Supporting Information: Supplementary Figure S1.. DOI: 10.1107/S2053230X16007202/rl5117sup1.pdf

## Figures and Tables

**Table:** Values in parentheses are for the highest resolution shell.

	SQFDLSTRRLKS (PDB entry 5icy)	(AcN)-CQFDLSTRRLRCGGSK (PDB entry 5icx)	(AcN)-CQFDLSTRRLKC-(Am) (PDB entry 5hpm)	CQFDLSTRRLKC-(Am) (PDB entry 5hyq)
Data collection				
Space group	*P*2_1_2_1_2_1_	*P*2_1_2_1_2_1_	*P*2_1_2_1_2_1_	*P*2_1_2_1_2_1_
Unit-cell parameters				
*a* (Å)	64.18	64.22	64.11	64.13
*b* (Å)	82.89	82.53	82.76	82.51
*c* (Å)	212.90	212.53	212.68	212.32
α = β = γ (°)	90.0	90.0	90.0	90.0
Resolution (Å)	34.82–2.50 (2.57–2.50)	33.00–2.60 (2.67–2.60)	33.00–2.60 (2.67–2.60)	32.53–2.48 (2.54–2.48)
Wilson *B* factor (Å^2^)	25.64	23.70	24.68	28.08
*R*_meas_	0.064 (0.245)	0.093 (0.385)	0.210 (0.986)	0.064 (0.270)
CC_1/2_	0.998 (0.951)	0.996 (0.915)	0.978 (0.539)	0.998 (0.928)
〈*I*/σ(*I*)〉	20.2 (5.9)	16.8 (4.7)	8.8 (1.9)	22.9 (5.4)
Completeness (%)	99.6 (96.1)	95.8 (93.7)	99.5 (100.0)	99.1 (88.9)
Multiplicity	4.7 (3.5)	4.4 (4.4)	4.4 (4.4)	5.1 (3.5)
Refinement				
Resolution (Å)	2.50	2.60	2.67	2.48
No. of reflections	40034	34159	32826	40613
*R*_work_/*R*_free_ (%)	16.8/21.6	17.7/22.2	18.7/23.2	16.6/20.8
No. of atoms				
Protein	6569	6561	6572	6569
Meditope	194	195	208	202
Water	501	341	279	501
*B* factors (Å^2^)				
Fab	20.9	21.1	29.2	24.1
Meditope	28.3	24.3	35.9	28.1
Water	25.3	24.0	29.7	29.8
R.m.s.d.				
Bond lengths (Å)	0.007	0.004	0.003	0.007
Bond angles (°)	1.095	0.798	0.619	0.899
Ramachandran statistics (%)				
Favored	96.9	97.1	97.1	96.9
Allowed	3.1	2.9	2.9	3.1
Disallowed	0.0	0.0	0.0	0.0

**Table:** 

	(MPT)QFDLSTRRLKC (PDB entry 5id1)	(AHA)QFDLSTRRLK (PDB entry 5id0)	AQFDLSTRRLKA (PDB entry 5esq)	GQFDLSTRRLKG (PDB entry 5icz)
Data collection				
Space group	*P*2_1_2_1_2_1_	*P*2_1_2_1_2_1_	*P*2_1_2_1_2_1_	*P*2_1_2_1_2_1_
Unit-cell parameters				
*a* (Å)	64.16	64.06	64.14	64.28
*b* (Å)	83.16	82.71	82.57	82.64
*c* (Å)	212.58	212.39	212.07	212.50
α = β = γ (°)	90.0	90.0	90.0	90.0
Resolution (Å)	34.43–2.49 (2.55–2.49)	34.74–2.48 (2.54–2.48)	34.26–2.55 (2.61–2.55)	29.66–2.55 (2.62–2.55)
Wilson *B* factor (Å^2^)	31.31	26.84	35.58	33.75
*R*_meas_	0.031 (0.138)	0.045 (0.209)	0.101 (0.482)	0.085 (0.365)
CC_1/2_	0.999 (0.984)	0.999 (0.963)	0.998 (0.850)	0.998 (0.893)
〈*I*/σ(*I*)〉	39.4 (11.0)	28.9 (7.2)	20.6 (3.9)	20.3 (4.5)
Completeness (%)	99.2 (92.5)	98.7 (89.6)	99.2 (93.0)	96.7 (91.5)
Multiplicity	7.8 (5.7)	5.9 (4.1)	5.5 (4.9)	5.1 (5.1)
Refinement				
Resolution (Å)	2.49	2.48	2.55	2.55
No. of reflections	40633	40600	37508	36560
*R*_work_/*R*_free_ (%)	17.3/22.1	16.0/20.8	19.0/24.2	17.1/22.1
No. of atoms				
Protein	6508	6553	6558	6550
Meditope	200	194	185	192
Water	419	490	352	390
*B* factors (Å^2^)				
Fab	22.1	17.7	31.8	27.5
Meditope	29.0	23.5	38.9	29.4
Water	24.8	21.4	33.1	30.2
R.m.s.d.				
Bond lengths (Å)	0.008	0.007	0.004	0.004
Bond angles (°)	1.191	1.139	0.757	0.803
Ramachandran statistics (%)				
Favored	97.2	97.5	97.2	96.9
Allowed	2.8	2.5	2.8	3.1
Disallowed	0.0	0.0	0.0	0.0

**Table 2:** Binding kinetics for meditope variants to cetuximab

Meditope	*k*_a_ (*M* ^−1^ s^−1^) × 10^4^	*k*_d_ (s^−1^)	*K*_d_ (n*M*)
CQFDLSTRRLKC	8.8	0.015	170
SQFDLSTRRLKS	0.31	0.027	8700†
(AcN)-CQFDLSTRRLRCGGSK	9.2	0.010	110
(AcN)-CQFDLSTRRLKC-(Am)	15	0.011	74
CQFDLSTRRLKC-(Am)	12	0.018	150
WSGGGCQFDLSTRRLRC	20	0.012	63
CQFDLSTRRLRCGGGSW	12	0.012	100
(MPT)QFDLSTRRLKC	2.1	0.012	560
(AHA)QFDLSTRRLK	3.8	0.064	1700‡
GQFDLSTRRLKG	1.7	0.083	5000‡
AQFDLSTRRLKA	5.8	0.091	1600‡

† Approximate value owing to poor fit quality and estimated concentration.

‡ The error in the *k*_a_ and *K*_d_ is estimated to be <38%. This is owing to the poor optical qualities of the different variants (see §[Sec sec2]2). However, the *k*_off_ values are independent of concentration.
