# Supplementary material for: Cyclization strategies of meditopes: affinity and diffraction studies of meditope–Fab complexes
Source: Acta Crystallogr F Struct Biol Commun. 2016 May 23;72(Pt 6):434–42. doi: 10.1107/S2053230X16007202 (PMC4909242; doi:10.1107/S2053230X16007202)
Supplement: Supplementary file 1 [file f-72-00434-sup1.pdf]

STRUCTURAL BIOLOGY COMMUNICATIONS

Volume 72 (2016)

Supporting information for article:

Cyclization strategies of meditopes: affinity and diffraction studies of meditope–Fab complexes

Krzysztof P. Bzymek, Yuelong Ma, Kendra A. Avery, David A. Horne and John C. Williams

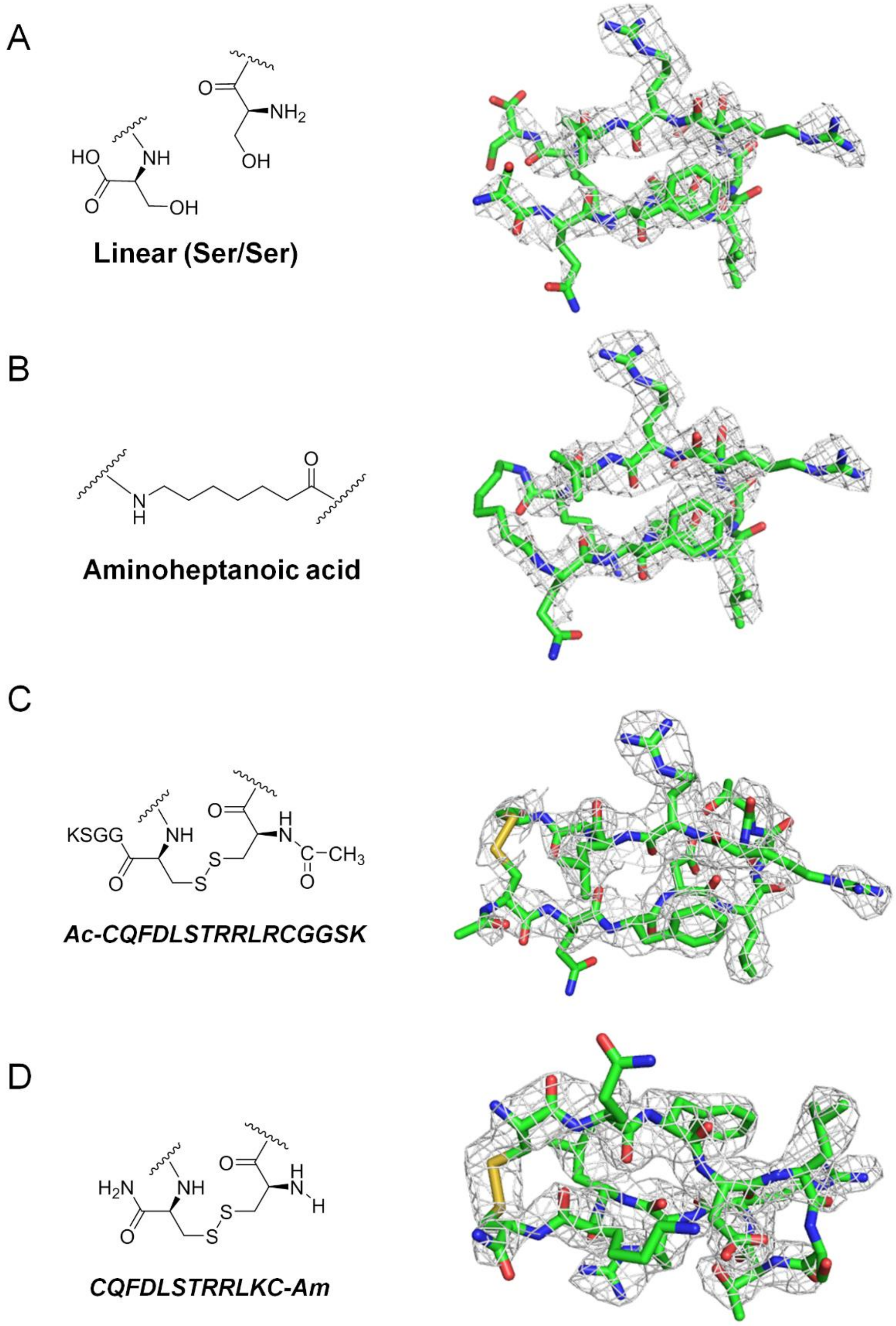
A
O
NH2
HO
NH
OH
O
OH
Linear (Ser/Ser)
B
O
N
H
Aminoheptanoic acid
C
O
H
KSGG
NH
N
C−CH3
S
O
S
O
Ac-CQFDLSTRRLRCGGSK
D
O
H2N
NH
H
N
S
H
O
S
CQFDLSTRRLKC-Am

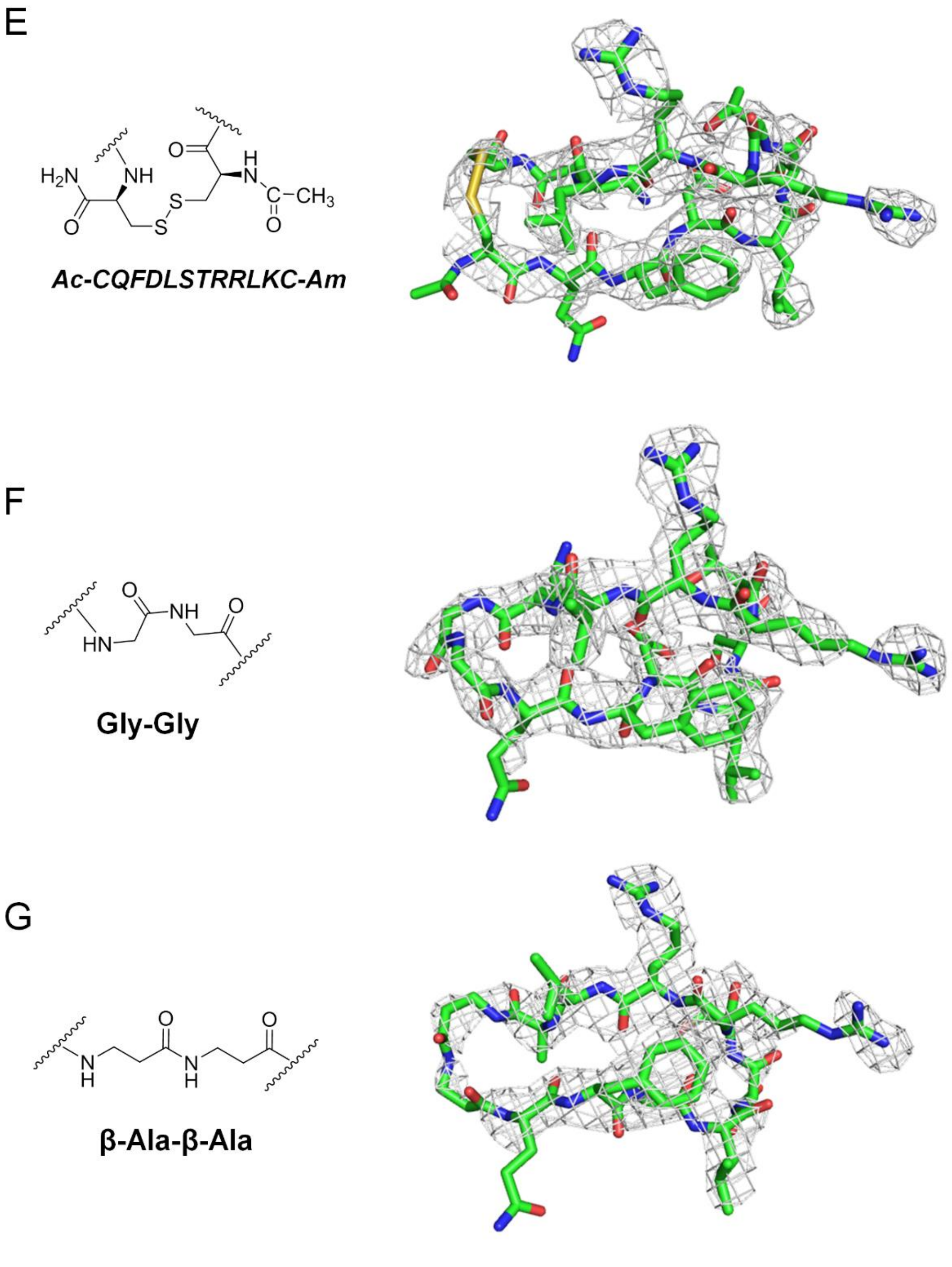

Figure S1 Fo-Fc omit maps calculated with Phenix for each of the meditope variants. Maps were contoured at 1σ. A) linear meditope; B) aminoheptanoic acid linker; C) long meditope; D) C-terminal amidated meditope; E) N-terminal acetylated, C-terminal amidated meditope; F) diglycine linked meditope F) di-β-alanine linked meditope.
